# A method for noninvasive individual genotyping of black‐footed cat (*Felis nigripes*)

**DOI:** 10.1002/ece3.11315

**Published:** 2024-04-23

**Authors:** Vimbai I. Siziba, Michelle M. Scroeder, Beryl Wilson, Alexander Sliwa, Sandi Willows‐Munro

**Affiliations:** ^1^ School of Life Sciences University of KwaZulu‐Natal Scottsville South Africa; ^2^ Black‐Footed Cat Working Group Kimberley South Africa; ^3^ McGregor Museum Kimberley Northern Cape South Africa; ^4^ Kölner Zoo AG (Cologne Zoo) Köln Germany

**Keywords:** allelic dropout, fecal DNA, *Felis nigripes*, genotyping error, microsatellites, scats

## Abstract

The black‐footed cat (*Felis nigripes*) is endemic to the arid regions of southern Africa. One of the world's smallest wild felids, the species occurs at low densities and is secretive and elusive, which makes ecological studies difficult. Genetic data could provide key information such as estimates on population size, sex ratios, and genetic diversity. In this study, we test if microsatellite loci can be successfully amplified from scat samples that could be noninvasively collected from the field. Using 21 blood and scat samples collected from the same individuals, we statistically tested whether nine microsatellites previously designed for use in domestic cats can be used to identify individual black‐footed cats. Genotypes recovered from blood and scat samples were compared to assess loss of heterozygosity, allele dropout, and false alleles resulting from DNA degradation or PCR inhibitors present in scat samples. The microsatellite markers were also used to identify individuals from scats collected in the field that were not linked to any blood samples. All nine microsatellites used in this study were amplified successfully and were polymorphic. Microsatellite loci were found to have sufficient discriminatory power to distinguish individuals and identify clones. In conclusion, these molecular markers can be used to monitor populations of wild black‐footed cats noninvasively. The genetic data will be able to contribute important information that may be used to guide future conservation initiatives.

## 1 INTRODUCTION

The black‐footed cat (*Felis nigripes* [Burchell 1824]) is the smallest wild cat species in Africa (Sliwa, 2013). Endemic to the arid region of southern Africa, the species has the most restricted distribution of any African felid (Sliwa, 2004; Sliwa et al., 2016). The species' core distribution is in South Africa although some populations are also found in Botswana, Namibia, and marginally in Angola and Zimbabwe (Wilson et al., 2016). Black‐footed cats are listed as Vulnerable by the International Union for Conservation of Nature (IUCN) due to limited range and small population sizes (Sliwa et al., 2016). Due to its elusive, solitary, and nocturnal habit, much of the black‐footed cat's ecology, behavior, and population dynamics remain unknown (Molteno et al., 1998; Sliwa, 1994, 2004, 2006).

Traditional methods of studying carnivores are challenging to implement for black‐footed cats because of low trapping success (Sliwa et al., 2010, 2018). Most studies of black‐footed cats have been based on direct observations (Sliwa, 1994, 2004, 2006) and camera traps (Sliwa et al., 2018). Direct observations are extremely time‐consuming and laborious as the species is highly mobile and travels long distances (Sliwa, 2004). Camera traps have been shown to have low efficacy for the species because individuals do not travel predictable routes marked by scent (Molteno et al., 1998; Sliwa, 2006). In rare cases where the cats have been recorded, they often move too fast for behavior to be captured by camera (Sliwa et al., 2018). Despite these challenges, direct observations have aided in answering questions about the relationships of black‐footed cats with other carnivores (Kamler et al., 2015), estimating home ranges and habitats (Sliwa, 2004), social organization and seasonal prey preferences (Sliwa, 1994, 2006). However, significant knowledge gaps still exist in the black‐footed cats' population estimates in the wild. To this end, DNA data could contribute toward the conservation of the species by identifying individuals and possible population estimates. Recent approaches such as metabarcoding have been used in diet studies in carnivores (Forin‐Wiart et al., 2018) and could also provide additional crucial ecological information on the feeding behavior of black‐footed cats.

A noninvasive approach to monitor populations of scarce species such as the black‐footed cat, could be more sustainable and reliable (Banks & Piggott, 2022; Piggott et al., 2008). DNA extracted from scats reduces the need to handle the animal (Ramon‐Laca et al., 2015) and could provide information on individual occupancy (Fernando et al., 2003), paternity and kinship (Constable et al., 2001; Wang et al., 2015), genetic diversity (Mengulluoglu et al., 2019), species identification and sex (Dalén et al., 2004; Kurose et al., 2005), identifying hybrids (Adams et al., 2003), and estimating population sizes (Banks et al., 2002; Eggert et al., 2003; Piggott et al., 2006). DNA amplification from scat material can be challenging as DNA may be degraded, leading to low amplification success (Ramon‐Laca et al., 2015; Taberlet et al., 1996) or may contain genotyping errors due to allelic dropout and false alleles (Fernando et al., 2003; Taberlet et al., 1996). Microsatellite loci are routinely used in the analysis of pedigree, genotyping, and parentage (Balloux & Lugon‐Moulin, 2002; Constable et al., 2001; Estoup et al., 1998; Reis et al., 2008; Vigilant et al., 2001), and have been used on scat samples in many studies (Bourgeois et al., 2019; Kurose et al., 2005). However, with felids most studies are biased toward large felids such as snow leopard (*Panthera uncia*) (Janečka et al., 2008) and leopard (*Panthera pardus*) (Mondol et al., 2009).

In this study, we test the utility of a suite of microsatellite markers as a molecular tool for the individual identification and monitoring of black‐footed cats using DNA extracted from scats. As well as to identify which microsatellite loci were the most informative with low genotyping errors. We compared the genotypes obtained from DNA extracted from scats to genotypes obtained from high‐quality DNA extracted from blood taken from the same individual to assess any possible error rates.

## 2 MATERIALS AND METHODS

### 2.1 Sample collection and DNA extraction

Blood and scat samples were collected from Benfontein Nature Reserve in Kimberley, Northern Cape Province, South Africa. Blood samples were taken from eight individual cats, four individuals had corresponding scat samples. These matched scat samples were collected while the cats were handled during radio‐collaring events. Two scats were collected per individual cat from which blood was collected, except for one cat where seven scat samples were collected. The rest (*n* = 70) were scat samples collected using a trained detection dog‐handler team (Long et al., 2007; MacKay et al., 2008). The training followed general guidelines recommended for conservation detection dogs (DeMatteo et al., 2019; Reed et al., 2011). Blood and scat samples were collected from March 3, 2020 to July 9, 2020 as part of an ongoing research project of the Black‐footed Cat Working Group (Sliwa et al., 2021). Samples were collected under the following permits: Northern Cape Department of Environment and Nature Conversation FAUNA 1218/2016, FAUNA 0636/2021. Blood samples were collected and stored onto Whatman FTA Elute (Sigma‐Aldrich, St Louis, Missouri, USA) cards. Scat samples were collected and stored in falcon tubes with 96% ethanol. Both blood and scat samples were placed in −80°C freezers for long‐term storage at the McGregor Museum, Northern Cape, South Africa.

DNA from blood samples was extracted using the E.Z.N.A® Tissue DNA kit (Omega BioTek, Georgia, USA) following the standard protocol. DNA from scat samples was extracted from 0.22 g of material scraped from the outer layer of each scat using the Qiagen QIAmp Fast DNA stool kit (Qiagen Inc., Hilden, Germany) following the standard protocol. All DNA extracts were stored at −20°C until PCR amplification of microsatellite loci.

### 2.2 Sex determination and microsatellite genotyping

All samples were sexed using a nested PCR protocol with the first reaction using the primers RG4 and RG7 (Griffiths & Tiwari, 1993) and the second PCR using Carni‐SRY2 and SRY‐CR1 primers (Kurose et al., 2005). The first PCR amplifications were performed using 35 cycles of denaturation at 94°C for 1 min, annealing at 50°C for 1 min, extension at 72°C for 1 min, and a final extension of 72°C for 10 min. The second PCR was the same as the first except the number of cycles was increased to 45. The PCR products were run on a 1% TBE agarose gel and visualized on the GelDoc™ EZ Imager (Biorad, Johannesburg, South Africa). The presence of a 135 bp band indicated the presence of the partial SRY gene and identified the individual as male. The absence of a band indicated the sample came from a female. If no band was present, the PCR was repeated with increased DNA concentration to confirm the absence of a band.

Nine unlinked autosomal feline microsatellites (Fca_08, Fca_23, Fca_26, Fca_48, Fca_58, Fca_88, Fca_126, Fca_132, and Fca_149) (Menotti‐Raymond et al., 1997; Menotti‐Raymond & O'Brien, 1995) were screened for use in this study. These markers were initially designed for domestic cats (Menotti‐Raymond et al., 1999) but have been used previously in black‐footed cats (Mattucci et al., 2018). All forward primers were labeled with a fluorescent dye on the 5′ end. Amplifications were performed in 12.5 μL reactions containing 6.25 μL TEMPase Multiplex Taq (Amplicon, Odense, Denmark), 4 μL dH_2_O, and 0.25 μL each of the forward and reverse primer. Amplifications were performed with an initial denaturation step at 94°C for 2 min, followed by 35 cycles of denaturation at 94°C for 15 s, annealing at 57°C for 30 s, extension at 68°C for 30 s and a final extension of 68°C for 5 min. Amplicons were sent to the Central Analytical Facility (CAF) at Stellenbosch University, South Africa for fragment analyses using an ABI PRISM™ 3500XL Genetic Analyzer (Life Technologies, Applied Biosystems, Warrington, UK) and a standard GeneScan™ ROX500™ (Applied Biosystems) internal size standard. The software package Geneious v8 (https://www.geneious.com) was used for genotype scoring. All scat samples were re‐genotyped up to five times through PCR amplifications and fragment analysis genotype scores were compared throughout to check for consistency. No samples were discarded for comparison purposes. To further validate the use of the microsatellite markers in black‐footed‐cat samples, each marker was used to perform a BLASTn search against the black‐footed cat genome (https://www.ncbi.nlm.nih.gov/datasets/genome/GCA_028533295.1/) in order to test for primer mismatches and to validate the consistency of the microsatellite mortif.

### 2.3 Data analyses

#### 2.3.1 Genotyping comparison: Blood versus scat

The genotypes recovered from blood and scat from the same individuals (*N* = 4) were compared for each microsatellite loci. The genotypes obtained from the same individual were scored as either matching (same genotype for blood and scat), not matching (different genotypes), or unreadable (either blood or scat had no readable data). The PCR success rate for each locus was assessed based on the total number of successful PCRs, allelic dropout, and false alleles through comparison between the blood and scat samples. We also compared null allele frequency, number of alleles, and the polymorphic information content (Piche et al., 2010) of each locus.

#### 2.3.2 Assessing the utility of microsatellite loci

The presence of null alleles was estimated using the expectation maximization (EM) algorithm (Dempster et al., 1977) in FreeNA (Chapuis & Estoup, 2007) for both blood and scat samples. The polymorphic information content (Piche et al., 2010) was estimated for each locus in Cervus v3.0.7 (Kalinowski et al., 2007). PIC values higher than 0.6 were considered highly informative (Mateesu et al., 2005). The minimum number of microsatellite loci needed to accurately identify individual cats was assessed by ranking loci by PIC values and systematically excluding loci with lower PIC values until the panel was no longer able to detect identity. Allelic richness (Ar) was estimated using the rarefaction method in FSTAT v2.9.3.2 (Goudet, 1995) to account for the difference in sample size between the blood and scat samples. The discriminatory power of each locus was assessed by calculating the probability of identity (P_ID_) and probability of exclusion (P_E2_‐ excluding a putative parent pair) for each locus in GenAlEx v6.503 (Peakall & Smouse, 2012). Overall P_ID_ and P_E2_ were also calculated using data from all loci. The P_ID_ was calculated using a conservative approach, assuming that a black‐footed cat was homozygous for the most common allele at each locus (Butler, 2005). Deviation from Hardy–Weinberg equilibrium (HW *p*‐value) was estimated in Cervus.

### 2.4 Individual identification

Assignment of scats to individual cats was done using GIMLET v1.3.3 (Valière, 2002) by identifying identical genotypes and comparing genotypes to a set of reference genotypes. GIMLET allows the selection of specific genotypes to be used as a reference to identify similar genotypes in the rest of the dataset, even when some loci are not amplified. This is particularly useful when using degraded DNA that does not always successfully amplify across all loci. Genotypes amplified from the blood samples were used as reference genotypes. As a compliment to the GIMLET analyses, principal coordinates analyses (PCoA) were conducted in GenAlEx (Peakall & Smouse, 2012), and identity analyses were conducted in Cervus (Kalinowski et al., 2007). The last two analyses do not take into account reference samples and can be biased by missing data.

Loci were ranked from the lowest to highest based on PIC values and the probability of identity was calculated for the entire loci set. Thereafter, the probability of identity was calculated repeatedly after excluding the marker with the lowest PIC value. The probability of identity was recalculated continuously excluding the lowest ranking PIC marker until only one marker remained.

## 3 RESULTS

### 3.1 Sample collection, DNA extraction, sex determination, and microsatellite genotyping

A total of 70 scat samples were collected in the field. Sixty‐six of these samples were found by detection dogs and the other four were identified as possible black‐footed cat scat by field staff. DNA from all the blood and scat samples were successfully extracted and all nine microsatellite loci were amplified. All the scat samples linked to blood samples were correctly identified as male or female. The rest of the field‐collected scat were identified as either male or female depending on the presence or absence of a 135 bp PCR product. The amplification success of individual loci varied even when amplified on high molecular weight DNA extracted from blood.

### 3.2 Blood versus scat genotypes

Genotypes produced from blood and scats collected from the same individual were compared for consistency. The sex of the individual was correctly determined for all scat and blood samples (Table 1). A comparison of heterozygosity indices found that scat samples generally showed reduced heterozygosity reflecting the impact of allelic dropout. Table 1 shows that the majority of the loci showed that corresponding scat samples had between 1 to 3 fewer alleles when compared with genotypes generated from blood. Loci Fca_43 showed an equal number of alleles between blood and scat samples, while three of the markers (Fca_26, Fca_58, and Fca_149) showed an increase in the number of alleles in the scat samples. Amplification success ranged from 87.5% (Fca_126) to 100% (Fca_43).

**TABLE 1:** Microsatellite loci comparison between black‐footed cat (*Felis nigripes*) blood and the corresponding scat samples showing missing data (allelic dropout [ADO]), null alleles, and incorrect genotypes (false alleles [FA]) across nine loci amplified.

Sample	Sex	Sample type	Missing data (ADO)	Null alleles in scat sample	Incorrect genotype reads in scat sample (FA)
Ca	Male	Blood	0.00	–	–
Sa	Female	Blood	11.11	–	–
Pu	Male	Blood	0.00	–	–
Te	Male	Blood	0.00	–	–
Du	Female	Blood	0.00	–	–
Du1	Female	Scat	44.44	11.11	33.33
Du2	Female	Scat	44.44	11.11	33.33
Ha	Male	Blood	0.00	–	–
Ha1	Male	Scat	11.11	0.00	0.00
Ha2	Male	Scat	0.00	11.11	11.11
Ph	Male	Blood	0.00	–	–
Ph1	Male	Scat	11.11	11.11	22.22
Ph2	Male	Scat	22.22	22.22	0.00
Ka	Female	Blood	0.00	–	–
Ka1	Female	Scat	22.22	0.00	22.22
Ka2	Female	Scat	44.44	0.00	22.22
Ka3	Female	Scat	44.44	0.00	22.22
Ka4	Female	Scat	22.22	11.11	33.33
Ka5	Female	Scat	11.11	55.56	22.22
Ka6	Female	Scat	22.22	33.33	33.33
Ka7	Female	Scat	22.22	44.44	33.33

*Note*: The “–” denotes genotypes where blood samples were not analyzed. Each blood and corresponding scat sample has a similar name. The scat sample has a numerical value attached to the name indicating how many scat samples were collected per individual cat.

### 3.3 Assessing the utility of microsatellite loci

Most markers, when analyzed for both blood and scat samples were highly informative, except for loci Fca_23, Fca_43, and Fca_58 (Table 2, see PIC values). When analyzing the scat samples the same markers (Fca_23, Fca_43, and Fca_58) together with markers Fca‐126 and Fca_132 were considered uninformative based on low PIC values. When using noninvasive samples five markers were highly informative and were sufficient to discriminate individual cats. Marker Fca_08, Fca_23, Fca_58, and Fca_88 had an equal number of alleles in both blood and scat samples. Marker Fca_126 and Fca_132 showed a lower number of loci in the scat samples when compared with the blood samples. Marker Fca_26 and Fca_43 showed one more allele in the scat samples than in the blood samples. Blood samples had an average of four alleles, compared to scat samples that had three (Table 2).

**TABLE 2:** Primer and loci details, BLASTn results, and genetic diversity estimates per locus from the free‐ranging black‐footed cat (*Felis nigripes*) data used in this study.

Locus	Primer sequence (5′–3′)	Motif	Primer mismatch	Chromosome	Amplification success (%)	Heterozygosity	PIC	Null allele frequency	NA	Ar
Fca_08	F‐ ACTGTAAATTTCTGAGCTGGCC R‐ TGACAGACTGTTCTGGGTATGG	CA	Nine	Seven	Blood: 100.00 Scat: 76.92	Blood: 0.75 Scat: 0.78	Blood: 0.71 Scat: 0.71	Blood: 0.10 Scat: 0.34	Blood: 5.00 Scat: 5.00	Blood: 5.44 Scat: 3.62
Fca_23	F‐ ACTGTAAATTTCTGAGCTGGCC R‐ TGACAGACTGTTCTGGGTATGG	CA	**–**	Three	Blood: 100.00 Scat: 84.62	Blood: 0.25 Scat: 0.33	Blood: 0.51 Scat: 0.16	Blood: 0.00 Scat: 0.07	Blood: 3.00 Scat: 3.00	Blood:3.70 Scat: 2.71
Fca_26	F‐ ACTGTAAATTTCTGAGCTGGCC R‐ TGACAGACTGTTCTGGGTATGG	CA		Twelve	Blood: 100.00 Scat: 92.31	Blood: 0.75 Scat:0.66	Blood: 0.67 Scat: 0.61	Blood: 0.00 Scat:0.00	Blood: 4.00 Scat: 5.00	Blood:3.74 Scat: 5.62
Fca_43	F‐ ACTGTAAATTTCTGAGCTGGCC R‐ TGACAGACTGTTCTGGGTATGG	CA	**–**	Five	Blood: 100.00 Scat: 100.00	Blood: 0.86 Scat: 0.77	Blood: 0.67 Scat: 0.53	Blood: 0.00 Scat:0.00	Blood: 4.00 Scat: 5.00	Blood:4.49 Scat: 4.12
Fca_58	F‐ ACTGTAAATTTCTGAGCTGGCC R‐ TGACAGACTGTTCTGGGTATGG	CA	–	Sixteen	Blood: 100.00 Scat: 92.31	Blood: 0.61 Scat: 0.29	Blood: 0.36 Scat: 0.15	Blood: 0.00 Scat:0.22	Blood: 2.00 Scat: 2.00	Blood: 3.74 Scat: 6.66
Fca_88	F‐ ACTGTAAATTTCTGAGCTGGCC R‐ TGACAGACTGTTCTGGGTATGG	CA	**–**	Seven	Blood: 100.00 Scat: 84.62	Blood: 0.93 Scat: 0.81	Blood: 0.79 Scat: 0.62	Blood: 0.00 Scat:0.00	Blood: 6.00 Scat: 6.00	Blood: 6.89 Scat: 4.49
Fca_126	F‐ ACTGTAAATTTCTGAGCTGGCC R‐ TGACAGACTGTTCTGGGTATGG	CA	–	Seven	Blood: 100.00 Scat: 76.92	Blood: 0.75 Scat: 0.54	Blood: 0.61 Scat: 0.39	Blood: 0.00 Scat: 0.08	Blood: 4.00 Scat: 2.00	Blood: 4.69 Scat: 2.00
Fca_132	F‐ ACTGTAAATTTCTGAGCTGGCC R‐ TGACAGACTGTTCTGGGTATGG	CA	**–**	Sixteen	Blood: 87.50 Scat: 84.62	Blood: 0.61 Scat: 0.42	Blood: 0.61 Scat: 0.32	Blood: 0.00 Scat:0.17	Blood: 4.00 Scat: 2.00	Blood: 3.00 Scat: 1.99
Fca_149	F‐ ACTGTAAATTTCTGAGCTGGCC R‐ TGACAGACTGTTCTGGGTATGG	AC	**–**	Three	Blood: 100.00 Scat: 61.54	Blood: 0.75 Scat: 0.51	Blood: 0.75 Scat: 0.72	Blood:0.00 Scat: 0.25	Blood: 5.00 Scat: 5.00	Blood: 4.44 Scat: 6.00

*Note*: PIC (Polymorphic information content), Null allele frequency, NA (Number of Alleles), and Ar (Allelic richness) values are given for blood and scat separately.

The locus allele frequencies for the blood samples ranged from 3.00 (Fca_132) to 6.89 (Fca_88). For the scat samples, allele frequencies ranged from 1.99 (Fca_132) to 5.62 (Fca_26) (Table 2). Only four loci showed null allele frequencies above 0.1 (Fca_08, Fca_58, Fca_132, and Fca_149). The mean number of alleles per locus ranged from two (Fca_43, Fca_58, Fca_126, and Fca_132) to six (Fca_88). Eight of the nine markers showed high levels of heterozygosity, only one loci (Fca_132) showed heterozygosity deficiency.

Performing a BLASTn search of the microsatellite primers against the black‐footed cat genome showed that all the nine microsatellite markers had a dinucleotide (CA)_n_ mortif. The loci were distributed across five of the 19 chromosomes. Namely, chromosome three (Fca_23 and Fca_149), chromosome five (Fca_43), chromosome seven (Fca_8 and Fca_88), chromosome 12 (Fca_26), and chromosome 16 (Fca_58, Fca_132). Only one of the primers had primer mismatches. Fca_08 mismatched to chromosome nine.

All the markers have 100% amplification success in the blood samples except Fca_132 which had an amplification success of 87.5% (Table 2). In the scat samples, except for Fca_46, none of the markers had a 100% amplification success. Amplification success for the rest of the eight markers in the scat samples ranged from 61.54% (Fca_149) to 92.31% (Fca_26 and Fca_58). Amplification success for the scat samples was above 70% for eight of the nine markers.

Heterozygosity values for the blood samples ranged from 0.25 (Fca_23) to 0.93 (Fca_88). For the scat samples, heterozygosity values ranged from 0.29 (Fca_58) to 0.81 (Fca_88). In the blood samples, marker Fca_23 was the only marker with low genetic diversity. In the scat samples, marker Fca_23 as well as Fca_58 had low diversity values. Heterozygosity values for blood samples were higher than those for scat samples except for markers Fca_08 and Fca_23 (Table 2).

The PID values ranged from 0.06 (Fca‐88) to 0.39 (Fca_58) for the blood samples and from 0.36 (Fca_88) to 3.97 (Fca_132) for scat samples (Table 3). PIDSib values ranged from 0.36 (Fca_88) to 0.61 (Fca_88) for the blood samples and from 0.40 (Fca_149) to 0.85 (Fca_58) for the cat samples.

**TABLE 3:** Probability of identity for unrelated individuals, probability of identity for siblings, and probability of exclusion between blood and fecal scats of black‐footed cat (*Felis nigripes*) across nine microsatellite loci.

Source	Microsatellite loci								
	Fca_08	Fca_23	Fca_26	Fca_43	Fca_58	Fca_88	Fca_126	Fca_132	Fca_149
P_ID_ Blood (*n* = 4)	0.10	0.25	0.13	0.13	0.39	0.06	0.17	0.17	0.08
P_ID_ Scat (*n* = 8)	1.22	2.31	0.55	1.19	0.36	1.49	3.26	3.97	0.70
PID_Sib_ Blood (*n* = 8)	0.40	0.52	0.42	0.42	0.61	0.36	0.46	0.46	0.38
PID_Sib_ Scat (*n* = 4)	0.41	0.84	0.46	0.52	0.85	0.46	0.60	0.66	0.40
P_E2_ Blood	0.68	0.28	0.68	0.77	0.52	0.83	0.68	0.52	0.52
P_E2_ Scat	0.68	0.52	0.87	0.77	0.90	0.77	0.28	0.28	0.83

**FIGURE 1:**
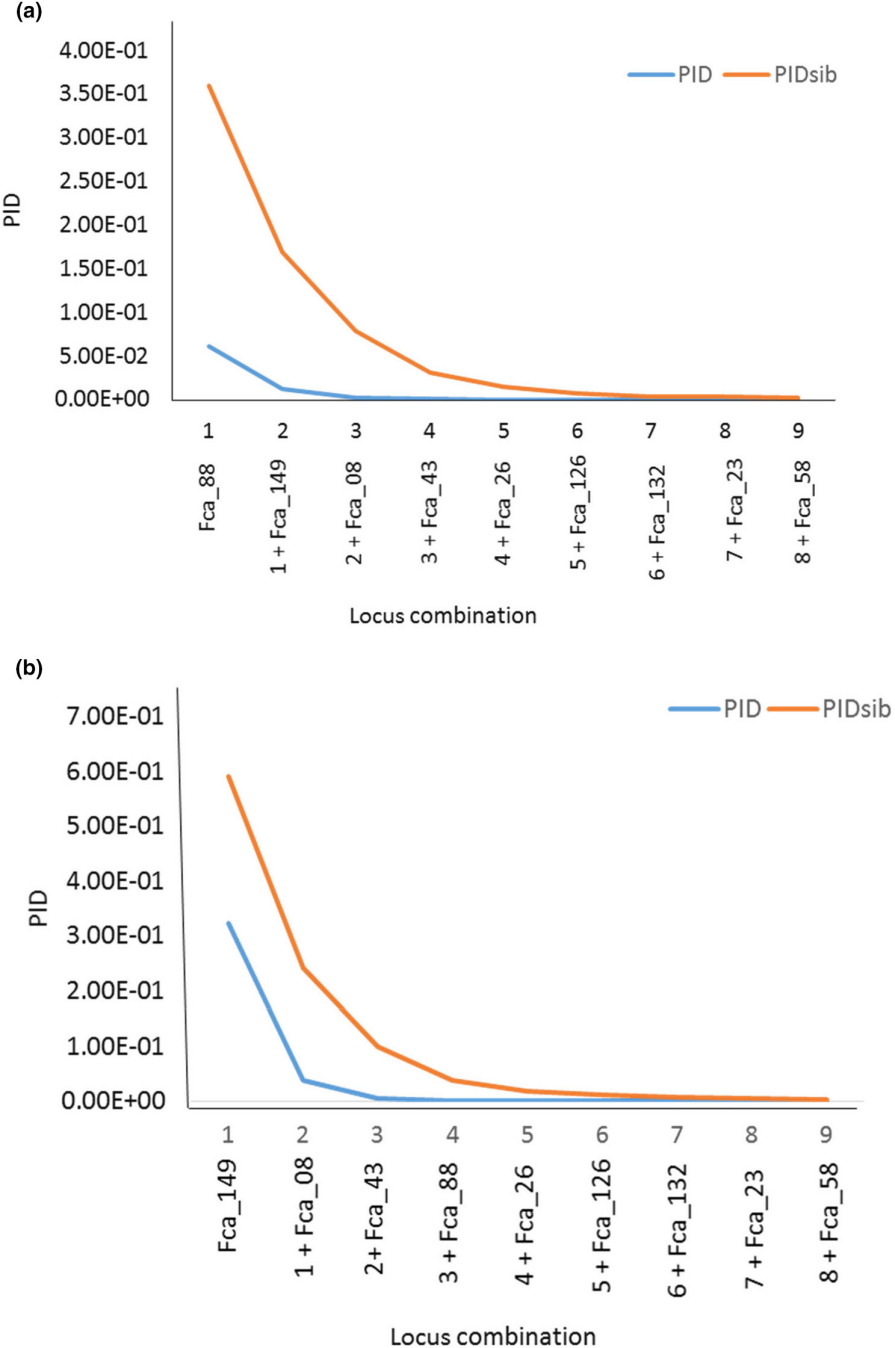
Probability of identity and probability of sib identity (a—Blood, b—Scat) for each locus and for increasing combinations for the nine loci. Each number represents the combination of the previous microsatellite locus combination.

For the blood samples, the highest ranking PIC marker was Fca_88, and for scat samples was Fca_149. Both blood and scat samples recovered Fca_58 as the locus with the lowest PIC lowest PIC marker was Fca_58. Plotting the PID values against the locus combinations shows that five markers are sufficient for the analysis of black‐footed cat individuals in both blood and scat samples (Figure 1). The combination of the five markers based on PIC was the same in both blood and scat samples (Fca_08, Fca_26, Fca_43, Fca_88, and Fca_149). The differences in blood samples (Figure 1a) and scat samples (Figure 1b) are the order in which the markers are excluded based on their PIC values (Table 1).

### 3.4 Individual identification

Analysis of the scat samples that were obtained by sniffer dogs showed that out of 70, 35 were identified as black‐footed cats based on their allele sizes. Individual analyses showed that the majority of the scat samples belonged to four individual cats already identified in the blood samples. An additional five cats, which were not in the reference genotype, were individualized based on their genotypes. Thirteen scat samples were assigned to the same blood sample as the cat identified as Kazi (Ka), 10 were assigned to the same blood sample as Hamba (Ha), and three of the scats were assigned to the same blood sample as Durga (Du). The rest of the scats were identified as six separate individuals.

## 4 DISCUSSION

This study investigated the possibility of individual identification of black‐footed cats through the use of microsatellite markers and DNA collected from scat samples in the wild. These data were validated by comparing how amplification success, heterozygosity, allelic dropout, and probability of identity of the microsatellite markers are affected using DNA collected from blood and scat samples from the same individual cats. The data indicate that scat samples are a reliable source of microsatellite genotyping as shown by similar studies that compared genotyping between blood and scat samples of American bison (*Bison bison*) (Forgacs et al., 2019), koala (*Phascolarctos cinereus*) (Schultz et al., 2018), red wolf (*Canis rufus*) (Adams & Waits, 2008), Mojave desert tortoise (*Gopherus agassizii*) (Mitelberg et al., 2019), common bottlenose dolphins (*Tursiops truncatus*) (Parsons, 2001), Asian elephants (*Elephas maximus*) (Fernando et al., 2003), and red deer (*Cervus elaphus*) (Valière et al., 2017).

A previous study on captive black‐footed cats using predeveloped microsatellite markers for domestic cats was done using hair tufts in a Polish zoo (Mattucci et al., 2018). Collecting hair or fresh scats, however, is not always feasible with wild populations (Giambattista & Gentile, 2018; Piggott et al., 2008). Unlike the previous study, this study validates the practicality of using the DNA from scats collected in the wild for individual analysis by comparing error rates between blood and scat samples. The less than 100% amplification success is most likely due to the age of the scats sampled. These data were validated through a study by Schultz et al. (2018), which showed that 70% of 14‐day‐old scats had high‐quality DNA for individual genotyping analyses. The majority of mismatches in the dataset resulted from allelic dropout, which has been identified in various studies as a drawback of using scat samples (Fernando et al., 2003; Johnson & Haydon, 2007).

Marker Fca_58, which had the maximum allele size (>200 bp), also showed the highest allelic dropout and lowest heterozygosity in scats, which further supports the assumption that DNA degradation was the main cause of lack of amplification. All of the cats identified as heterozygotes using the blood samples for this marker (Fca_58), were identified as homozygotes using the scat samples. These data correlate with studies that have shown that longer alleles of heterozygous loci tend to have higher levels of allelic dropout in scat samples as a result of degraded DNA (Gerloff et al., 1995; Goossens et al., 1998). When used for population structure and genetic diversity, this marker will greatly underestimate the level of genetic diversity (Gagneux et al., 1997; Pompanon et al., 2005; Waits & Leberg, 2000). Loci with shorter amplicons have higher amplification and lower error rates and are therefore more ideal for scat samples (Broquet et al., 2006; Frantzen et al., 1998). Markers Fca_23 and Fca_58 showed the lowest PIC values. This means in downstream applications, they are the first markers to be excluded when analyzing paternity, kinship, or even the identification of an individual. For the other markers with low heterozygosity or high null allele values, the data were improved by repeating the PCRs. This study showed a loss in heterozygosity between blood and scat samples. These data correlate with other studies that have compared genotypes between blood and scat samples (Forgacs et al., 2019; Johnson & Haydon, 2007). However, the higher rates of genotyping failure in this study could be due to a significantly small sample size and the unavailability of scat samples for all the corresponding blood samples.

BLASTn results showed that the primer motif was conserved across both species of the domestic cat and black‐footed cat. Marker Fca_08 was the only marker that had a mismatch and it was also the only marker that had a null allele value higher than 0.00 in the blood samples. Primer mismatches have been shown to decrease the thermal stability of primer‐template matches thus reducing the PCR yield significantly (Kwok et al., 1999; Stadhouders et al., 2010). Primer mismatches were attributed to only one of the markers, therefore null alleles in the scat samples could be a result of low DNA yields as a result of DNA degradation in the field (Manning et al., 2022).

A comparison of null alleles between blood and scat genotypes showed that markers Fca_26, Fca_43, and Fca_88 had a zero null allele value in both blood and scat individuals. Markers Fca_23, Fca_126, and Fca_132 had low null allele frequencies. These six markers would be the better choice for population genetics studies as compared to the other markers that have higher null alleles. Black‐footed cats have low population sizes and are endemic to Southern Africa, unlike other wildlife species. Therefore, these markers would have a higher probability of identity when compared with other species with larger populations. Null alleles can result from low DNA template quality, mutations in the flanking region, or differential amplification of size variant alleles. The source of null alleles can be assessed through the comparison of alleles (Chapuis & Estoup, 2007; Dakin & Avise, 2004) and sequencing of the primer annealing sites (Callen et al., 1993).

Identifying individuals using blood samples showed that seven markers were highly informative (PIC value higher than 0.6). Analysis of the scat samples however, showed that only five markers were highly informative. This would be a direct result of allelic dropout in those markers that would result in a reduced PIC value. This is to be expected from noninvasive samples. This study therefore provides an accurate guideline on the best markers to use when studying noninvasive samples. Other studies have shown that as few as three markers were sufficient to discriminate individuals accurately (Parsons, 2001).

Individual identification of black‐footed cats using the older popular programs such as Cervus (Kalinowski et al., 2007) and Colony (Jones & Wang, 2010) may greatly overestimate the number of individual cats. This is because these programs do not take into account errors introduced by allelic dropout through direct comparison and were not ideal for the noninvasive genetic samples used in this study. GIMLET (Valière, 2002) showed the best results when used across the same individuals that had both blood and scat samples. This is mainly because GIMLET is adapted to analyzing genotypes obtained from noninvasive samples and accommodates error rates between the reference genotypes (blood samples) to the genotypes required for analysis (scat genotypes).

This study highlights the need to assess markers in low‐quality samples before use in scat samples with a larger dataset, especially in the case of endangered or threatened species where a noninvasive approach is ideal. Once these markers are verified, as is the case with this study, they can be used in other studies, such as population structure and genetic diversity (Schwartz et al., 2007). A paired sample‐based genotyping method such as this one provides the most accurate validation of scat DNA markers and other low‐quality DNA sources such as hair. Amplification was possible from all the nine markers used, however, some markers amplified better than others. Marker performance can be affected by various reasons, such as the allelic range, DNA degradation, or PCR artifacts. In most cases, the true cause of reduced marker performance cannot be accurately determined. As such, an assessment of markers in scat DNA to show which markers are most reliable is necessary before using the markers in larger datasets. This improves the estimation of allelic dropout and false allele error rates through a reference dataset (Johnson & Haydon, 2007).

This study, like other studies, shows that without careful assessment of markers skewed population estimates may be reported (Forgacs et al., 2019). However, because studies involving rare or endangered species rely on noninvasive samples, it is prudent to use studies such as this one to verify the reliability of each marker used and possibly select only the most reliable markers. This greatly reduces skewed estimates of genetic diversity (Taberlet et al., 1996). Our pilot study can be used as a reference and to monitor the growth of the black‐footed cat population in the Benfontein area. Similar studies have been used for monitoring brown bear (*Ursus arctos*) populations after reintroduction in the Italian Alps (De Barba et al., 2005).

## AUTHOR CONTRIBUTIONS

**Vimbai I. Siziba:** Conceptualization (equal); data curation (equal); formal analysis (equal); investigation (equal); writing – original draft (lead). **Michelle M. Scroeder:** Resources (supporting); writing – review and editing (equal). **Beryl Wilson:** Resources (supporting); writing – review and editing (supporting). **Alexander Sliwa:** Writing – review and editing (supporting). **Sandi Willows‐Munro:** Conceptualization (lead); formal analysis (supporting); funding acquisition (lead); investigation (supporting); methodology (supporting); project administration (lead); resources (supporting); supervision (lead); writing – review and editing (supporting).

## CONFLICT OF INTEREST STATEMENT

The authors have no conflict of interest in regard to this work.

## Data Availability

All the data used in this research are available within the manuscript and additional data are available on figshare.
